# Uncertainty Propagation in Nerve Impulses Through the Action Potential Mechanism

**DOI:** 10.1186/2190-8567-5-3

**Published:** 2015-01-12

**Authors:** Aldemar Torres Valderrama, Jeroen Witteveen, Maria Navarro, Joke Blom

**Affiliations:** CWI, Amsterdam, Science Park 123, 1098 XG Amsterdam, The Netherlands; Department of Neurology and Neurosurgery, Brain Center Rudolf Magnus, University Medical Center Utrecht, Heidelberglaan 100, 3584 CX Utrecht, The Netherlands

**Keywords:** Neurodynamics, Uncertainty Quantification, Sparse grid quadrature, Hodgkin–Huxley model, Neuronal Noise

## Abstract

We investigate the propagation of probabilistic uncertainty through the action potential mechanism in nerve cells. Using the Hodgkin–Huxley (H-H) model and Stochastic Collocation on Sparse Grids, we obtain an accurate probabilistic interpretation of the deterministic dynamics of the transmembrane potential and gating variables. Using Sobol indices, out of the 11 uncertain parameters in the H-H model, we unravel two main uncertainty sources, which account for more than 90 % of the fluctuations in neuronal responses, and have a direct biophysical interpretation. We discuss how this interesting feature of the H-H model allows one to reduce greatly the probabilistic degrees of freedom in uncertainty quantification analyses, saving CPU time in numerical simulations and opening possibilities for probabilistic generalisation of other deterministic models of great importance in physiology and mathematical neuroscience.

## 1 Findings

To provide sensible mechanistic models of neuronal circuits in the brain and to facilitate principled interpretations of data from experiments in vitro and in vivo, biophysical models of neurodynamic systems must incorporate variability as a defining feature [1–6]. Neuronal variability cannot be accounted for by dynamic models formulated as *deterministic* differential equations. However, such models can be extended to do so by incorporating *probabilistic degrees of freedom*. For this purpose, the versatile technique of Stochastic Collocation on Sparse Grids (SCSG) [7–9], from Uncertainty Quantification (UQ) theory [7, 10–13], has been recently suggested as a computationally efficient strategy [14].

In this report, we deploy SCSG to investigate uncertainty propagation through the action potential mechanism in the H-H model [15], namely
1

with , where  is the electrical potential across a neuron’s membrane, ,  are the gating variables associated with the activation and inactivation of  ion currents, respectively,  is the gating variable associated with the activation of  ions current and where *I* represents current injected through the cell membrane.

The ODE system (1) exhibits a combination of excitability and highly nonlinear dynamics. We will show how these features can render the model output extremely sensitive to parameter fluctuations under realistic physiological conditions, demonstrating that Uncertainty Quantification might be indispensable to provide sensible biophysical models of nerve impulses. The H-H model [15] includes a vector of 11 parameters, which in state space form reads
2

consisting of four initial conditions , , , , three parameters describing the maximum conductances , , , corresponding to ,  and leakage ion currents respectively, three parameters describing their equilibrium Nernst potentials , , , and the membrane capacitance *C*. These parameters are uncertain, for they must be determined experimentally and are therefore subject to errors and fluctuations. Their nominal values measured in squid axon preparations are listed in Table 2 [15].

In the probabilistic generalisation of the H-H model, a Banach space describing such 11 probabilistic degrees of freedom is required for uncertainty analysis. Without appropriate discretisation and dimensionality reduction procedures, the computational cost of such analysis depends exponentially on the number of parameters, challenging the capacities of current hardware.

An efficient numerical strategy to deal with such a curse of dimensionality, is to find a selection of points in probability space that yields a good approximation of the model’s response surface. This can be achieved by constructing a Sparse Grid in the multi-dimensional probability space for each time point following Smolyak’s algorithm [8, 9, 16–18].

Using Sparse Grid quadrature in conjunction with Sobol indices, we unravelled the parameters whose fluctuation causes most of the observed variability in neuronal responses. Such an analysis hinted at a parsimonious H-H model with two probabilistic degrees of freedom instead of 11, the use of which can achieve tremendous savings in CPU time as discussed below.

**Fig. 1:**
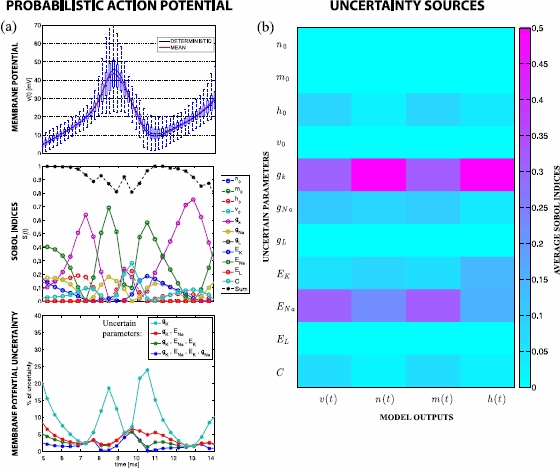
**a** *The top panel* shows the probabilistic membrane potential, distributions are indicated by box-and-whiskers plots at each time instant. *The middle panel* shows the first order Sobol indices, revealing the sources of the fluctuations in the membrane potential for each uncertain parameter. *The black line* is the sum of all indices; deviation from one indicates variance due to parameter interactions. *The bottom panel* shows the variance difference w.r.t. the model with 11 uncertain parameters for four parsimonious models. Only two parameters ( and ) are required to estimate a variability with less than 10 % error. **b** Average of the Sobol indices for all uncertain parameters during the time interval shown in **a** for the membrane potential and gating variables

The uncertain dynamics of the membrane potential during neuronal discharge is shown in Fig. 1(a). The probability distribution at each time point was obtained by Monte Carlo sampling of a third level Sparse Grid piecewise linear interpolant [8, 19] of the response surface for the H-H model, using 1000 samples per time point from a uniform probability distribution and assuming 20 % of variability in the nominal values of the parameters listed in Table 2. The mean and deterministic solutions are also shown. Notice that they differ when the membrane depolarisation reaches its acme, which is often a signature of high nonlinearity.

In Fig. 1, the mean was obtained at the fourth level of a Sparse Grid constructed using the Gauss–Patterson (GP) quadrature rule, which requires 18591 function calls. These deterministic sequences are nested by construction, and for smooth integrands they can achieve the maximum degree of exactness from all nested rules [9, 18, 20].

In Fig. 1 neuronal variability is reported on the top panel by box-and-whisker plots, with one box per time point. On each box, the central mark is the median, the edges of the box are the 25th and 75th percentiles and the whiskers extend to the most extreme membrane potentials observed.

The middle panel of Fig. 1(a) shows the dynamics of the first order Sobol indices [18, 21–24], computed by Sparse Grid quadrature. The Sobol indices in Fig. 1(a) allow one to determine the relative contribution of each uncertain parameter to the variability of the model output observed in the top panel at each instant in time. The black line shows the sum of all indices; deviation from one indicates fluctuations in the model output due to parameter interactions not accounted for by the first order indices.

The variance and Sobol indices were obtained at the third level of a GP Sparse Grid in 22 dimensions, requiring 17249 function calls. Notice that only 21 dimensions are needed to obtain the first order Sobol indices via the conditional variance. However, adding one extra dimension allows one to estimate the total variance with the same Sparse Grid, which often increases accuracy [18].

The RMS errors for the mean, variance and Sobol indices shown in Fig. 1 are summarised in Table 1. The most accurate numerical solution is taken as a reference for error analysis, thus the error estimates listed in the table are conservative, since they approximate the numerical error at the last but one quadrature level. Table 1 shows that the numerical errors are sufficiently small, validating the choice of quadrature level for further analysis.

**Table 1:** **RMS error of the Sobol indices show in Fig.** 1 **(a) for all model outputs**

	*E*	*σ*											
*n*(*t*,***ξ***)	0.000163	0.000350	0.016969	0.016969	0.016710	0.016970	0.012563	0.009419	0.016891	0.015160	0.011181	0.016949	0.015736
*m*(*t*,***ξ***)	0.001494	0.004814	0.090669	0.090668	0.089543	0.090673	0.097756	0.057230	0.090059	0.078554	0.062516	0.090535	0.082084
*h*(*t*,***ξ***)	0.000112	0.000271	0.046205	0.046205	0.045488	0.046202	0.035574	0.027616	0.045989	0.042473	0.017223	0.046155	0.042037
*v*(*t*,***ξ***)	0.004669	0.052106	0.087004	0.087002	0.084896	0.086993	0.079195	0.055766	0.086538	0.073698	0.060872	0.086917	0.078468

In Fig. 1(b) all uncertainty sources are represented in a colour map, showing the average value of all Sobol indices during the time interval shown in Fig. 1(a). All outputs of the model, namely the membrane potential and the three gating variables are shown.

Interestingly, this colour map reveals that only a small subset of parameters causes most of the uncertainty in the model outputs. Therefore, all other parameters can be fixed to their nominal values preserving the probabilistic interpretation of the model. This is illustrated in the bottom panel of Fig. 1(a), which shows the difference in standard deviation of four effective models with respect to that of the model with the 11 uncertain parameters listed in Table 2. Notice that only two parameters,  and , are required to estimate a variability with less than 10 % difference w.r.t. the original model for all time points.

**Table 2:** **Normalised ranking of the average Sobol indices displayed in the colour map in Fig.** 1 **(b) using SVD**

	Mode 1	Mode 2	Mode 3	Mode 4
Mode energy:	**0.944690**	0.042433	0.011498	0.001379
Nominal values				
	0.001064	0.000372	0.012061	0.034643
	0.001064	0.000372	0.012060	0.034648
	0.140569	0.436071	0.555783	1.000000
	0.001206	−0.000072	0.013534	−0.059476
	**1.000000**	−0.627724	−0.197631	0.384254
	0.172271	0.300390	−0.305118	0.084833
	0.001741	0.001124	0.016312	−0.091209
	0.232445	−0.225011	1.000000	−0.978672
	**0.532895**	1.000000	−0.190240	−0.759104
	0.001293	−0.000515	0.013721	−0.048586
*C* = 1 μF/cm^2^	0.099635	0.342083	0.410624	0.931327

**Fig. 2:**
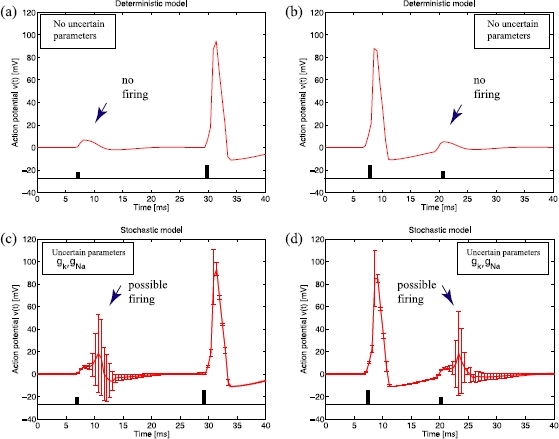
In panel **a** two current pulses are applied to a deterministic neuron with the nominal values for the parameters listed in Table 2. The first pulse is not strong enough to elicit a spike, the second stronger pulse immediately triggers a spike. Panel **c** shows the probabilistic counterpart of this experiment assuming 20 % of variability in the nominal parameters  and . *Large error bars* show that the first small current pulse can trigger action potentials in this instance. In panels **b** and **d** the stronger current pulse is applied first, immediately eliciting a spike. In the deterministic model shown in panel **b**, the second small pulse fails to trigger a spike when applied during the refractory period. In the probabilistic model **d**, large variability in the output indicates a second action potential, which would not be expected from deterministic predictions

The symbol ^∗^ indicates the initial values used in Fig. 2.

The singular value decomposition (SVD) of the  data matrix *M* displayed in Fig. 1(b) as a colour map, namely , provides a quantitative ranking of the average Sobol indices. The first four columns of *U* after normalising the patterns so that each column has maximum entry 1 are shown in Table 2. Notice that the first mode (first column of *U*) contains more than 90 % of the energy, given by the square of the singular values. Notice also that in this maximum energy mode, two probabilistic degrees of freedom are dominant, namely  and . The influence of all the other parameters in the variability of the model output is small. Therefore, they can be fixed to their nominal values. This greatly reduces the dimensionality of the model retaining most of its probabilistic features.

This feature of the H-H model is important, since it shows that probabilistic neurodynamic simulations can be performed parsimoniously, in an effective probability subspace of dimension far smaller than that of the original model. *This greatly reduces the computational cost and facilitates the analysis of neuronal systems beyond individual cells*.

As a non-trivial illustrative application of our parsimonious model Fig. 2 shows the impact of parameter fluctuations on two relevant neurocomputational properties, namely, neural response to current input and membrane refractoriness.

In Fig. 2(a) two 1 ms current pulses are applied to a deterministic H-H neuron whose membrane potential is initially at the resting potential. The first pulse of 8.5 μA/cm^2^ increases the membrane potential, but it does not elicit neuronal discharge. A second stronger pulse of 20 μA/cm^2^ applied later, immediately triggers a spike. The probabilistic counterpart of this experiment is shown in (c). The large error bars in response to the first pulse show that a small current pulse can indeed trigger action potentials contrary to deterministic predictions.

In panels (b) and (d) the strong current pulse of 20 μA/cm^2^ is applied first, immediately eliciting a spike. In the deterministic model shown in panel (b), a second small pulse of 10.5 μA/cm^2^ fails to ignite an action potential due to membrane refractoriness. Under parameter uncertainty, panel (d) shows large variability in the neuronal response, indicating a second action potential, which is impossible on deterministic grounds.

Synaptic input responses and membrane refractoriness are neurocomputational properties of realistic nerve cells, which directly influence collective neuronal behaviour as well as the efficiency of neural codes. Thus, the examples above illustrate how incorporating variability in neurodynamic models might be crucial to our understanding of the nervous system and the behaving brain.

Probabilistic generalisation of other deterministic models of great importance in mathematical neuroscience might be possible using the methods and results in this report, such as those describing neuronal interactions and signal transmission in active neural media, at a feasible computational cost.
